# 基于修饰组和探针分子的重要途径代谢物筛选和注释新方法

**DOI:** 10.3724/SP.J.1123.2022.03025

**Published:** 2022-09-08

**Authors:** Zaifang LI, Fujian ZHENG, Yueyi XIA, Xiuqiong ZHANG, Xinxin WANG, Chunxia ZHAO, Xinjie ZHAO, Xin LU, Guowang XU

**Affiliations:** 1.中国科学院大连化学物理研究所, 中国科学院分离分析化学重点实验室, 辽宁省代谢组学重点实验室, 辽宁 大连 116023; 1. CAS Key Laboratory of Separation Science for Analytical Chemistry, Liaoning Province Key Laboratory of Metabolomics, Dalian Institute of Chemical Physics, Chinese Academy of Sciences, Dalian 116023, China; 2.中国科学院大学, 北京 100049; 2. University of Chinese Academy of Sciences, Beijing 100049, China

**Keywords:** 液相色谱-高分辨串联质谱, 修饰代谢组, 次生代谢物, 探针分子, 注释, liquid chromatography-high resolution tandem mass spectrometry (LC-HRMS/MS), modified metabolome, secondary metabolites, probe molecule, annotation

## Abstract

植物次生代谢物在抵御生物/非生物胁迫、生物间互作以及信息传递等方面发挥重要作用,次生代谢途径解析对植物分子育种、天然产物合成等方面具有重要意义。液相色谱-高分辨串联质谱(LC-HRMS/MS)为次生代谢物鉴定及途径表征提供了技术手段。非靶向LC-HRMS/MS方法可获得丰富的质谱信号,包括一级质谱和二级质谱(MS, MS/MS),但受质谱数据库规模以及次生代谢物复杂性的制约,次生代谢物注释十分困难。该研究以玉米叶片中苯丙烷途径代谢物为例,发展用于非靶向代谢组数据中重要途径代谢物的高效筛选和注释新方法。首先,利用公共代谢途径数据库及文献获取参与苯丙烷代谢途径的61种修饰反应类型,进而从非靶向实验数据中筛选出修饰代谢组。其次,获取开源串联质谱数据中的苯丙烷类化合物作为探针分子,构建探针分子质谱数据库。将探针分子与修饰代谢组共建分子网络,锁定目标途径代谢物并注释结构。该方法在正、负离子模式下分别筛选出玉米叶片中392个和417个苯丙烷途径候选代谢物,去冗余后共注释出129个代谢物,涉及苯丙烷代谢的主要分支途径,如黄酮途径的8个类黄酮、19个氧苷类黄酮和32个碳苷类黄酮,31个羟基肉桂酸途径代谢物以及22个木脂素途径代谢物;其中26个在PubChem和SciFinder数据库中未见收录。该研究利用探针分子结合修饰组可快速锁定途径代谢物,且有助于快速、准确的网络传播注释,可显著提高目标途径代谢物筛选与注释效率,为植物次生代谢途径的深入解析提供分析手段。

## Abstract (en)

Plants produce a wide variety of secondary metabolites in the process of evolution. Secondary metabolites have highly diverse structures due to the modification of the basic skeletons of metabolites. They are required for interaction with the environment and are produced in response to abiotic/biotic stress. Characterization of secondary metabolic pathways is significant to plant molecular breeding and natural product biosynthesis. The liquid chromatography-high resolution tandem mass spectrometry (LC-HRMS/MS) is one of the major techniques for untargeted metabolomics study. The LC-HRMS/MS method could detect tens of thousands of metabolic features and provide abundant structural information. It has been widely used in the discovery and characterization of the secondary metabolome. However, due to the largely diverse structure and limited records in the mass spectral library, the annotation of the secondary metabolome is very difficult.

To address the analytical challenges associated with the vast structural diversity and the large numbers of secondary metabolites, particularly those previously unknown structural metabolites, a novel method for the efficient characterization of pathway-associated metabolites was developed. Modification reactions and MS/MS spectral information were collected using the metabolic pathways database and mass spectral library. Screening and annotation of metabolites involved in phenylpropanoid metabolism in maize leaves were used as an example. First, a database of modified groups was established via pathway-associated modifications from open access metabolic pathway database and literature. Here, pathway databases included the Kyoto Encyclopedia of Genes and Genomes (KEGG) and Plant Metabolic Pathways (PlantCyc). A total of 61 modification types were enrolled, including 10 generic and 51 pathway-specific modifications. Modified metabolomes were filtered from untargeted LC-HRMS/MS metabolomics data. Next, MS/MS spectra of the pathway-associated compounds (probe molecules) were collected in the Global Natural Products Social Molecular Networking (GNPS) MS/MS spectral library. The MS/MS of compounds assigned to chemical classes of phenylpropanoids were kept. An MS/MS spectral database of the probe molecules was constructed. It included 2677 spectra of 1542 phenylpropanoid compounds in the positive mode and 814 spectra of 661 phenylpropanoid compounds in the negative mode. Then, an MS/MS molecular network was generated by modified metabolome and probe molecules. The clusters comprising both probe molecules and modified metabolites were kept. To explore more previously unknown structural metabolites, the clusters with one more pathway-specific modified metabolite were retained even though they didn’t contain any probe molecule. A total of 392 and 417 phenylpropanoid pathway-related metabolic metabolites were obtained in positive and negative ion modes, respectively. The pathway-associated metabolites were annotated based on the propagation of the molecular network. For the metabolites within the co-cluster, annotations were performed using the probe molecules as the initial seed. The modification group’s substructure information was used for network propagation annotation. For the clusters containing only pathway-specific modified metabolites, the annotation is similar to the above process if identified nodes were present within the cluster. Otherwise, de novo annotation was manually executed based on substructure information. Finally, 129 unique metabolites were annotated after integration and removal of redundancy. Ten annotated metabolites were validated using commercially available or synthesized reference compounds. The other annotation results were validated using predicted chemical classes, *in silico* MS/MS, and predicted retention time. They are mainly involved in the downstream branch of phenylpropanoid pathways, including the flavonoid pathway (8 flavonoids, 19 flavonoid *O*-glycosides, 32 flavonoid *C*-glycosides), the hydroxycinnamic acid pathway (31 hydroxycinnamic acids and derivatives), and the lignan pathway (22 neo-lignans/lignan/lignan glycosides). All the annotated structures were searched against the PubChem and SciFinder databases. Among them, 26 metabolites were previously unreported in both the databases. In this study, the pathway-associated metabolites could be quickly discovered and annotated by the integration of probe molecules and modified metabolome. It provides a method for the in-depth study of the phenylpropanoid pathway.

植物在进化进程中,为适应复杂的生存环境及满足发育需要,产生出种类繁多、数量巨大、结构非常丰富的次生代谢物,在抵御生物/非生物胁迫、生物间互作以及信息传递等方面发挥重要作用^[1]^,次生代谢途径解析对植物分子育种及天然产物生物合成等具有重要意义。苯丙烷代谢是植物最重要的次生代谢合成途径之一,与植物应激诱导有着密切关系^[2,3]^。苯丙烷代谢有多个分支途径,如黄酮途径、木质素途径、木脂素途径、羟基肉桂酸酰胺途径等;骨架结构经多种后修饰,如羟基化、糖基化、乙酰化、异戊二烯化、硫酸化和甲基化等,产生了复杂多样的苯丙烷途径代谢物。

基于液相色谱-高分辨串联质谱(LC-HRMS/MS)的代谢组学分析技术为次生代谢物鉴定及途径阐释提供了手段^[4⇓⇓-7]^。如Wen等^[8]^基于非靶向LC-HRMS/MS代谢组学技术鉴别了玉米中29个类黄酮,通过整合基因组学、转录组学信息,揭示了玉米黄酮生物合成的遗传学基础。牟红梅等^[9]^基于超高效液相色谱-串联质谱的非靶向代谢组学分析研究了成熟期茄梨和红茄梨果皮代谢物差异,发现差异代谢物涉及黄酮代谢、氨基酸代谢、苯丙烷代谢以及苯丙烷分支途径黄酮代谢等。胡永丹等^[10]^基于超高效液相色谱-高分辨质谱联用技术分析茶树花化学成分,采用氮规则、质量亏损和特征子离子筛选目标化学成分,从7个茶树花样本中共鉴定出137个化合物。非靶向代谢组学采集了非常丰富的质谱信号,包括一级质谱和二级质谱(MS & MS/MS),代谢组鉴定多采用质谱数据库搜索方式^[11]^。尽管全球天然产物社会分子网络(Global Natural Products Social Molecular Networking, GNPS)数据库已收录免费共享的83314张MS/MS谱图,但与复杂多样的植物代谢组,特别是次生代谢组相比,质谱库的覆盖范围仍不足,搜库鉴定能力有限^[12]^。利用非靶向代谢组实验数据,构建基于质谱相似性(MS/MS similarity)的分子网络(molecular networking, MN)^[13,14]^,可人工推断注释数据库无法鉴定的结构类似物^[15]^。如Clements等^[16]^采用代谢组学结合分子网络的方法,推测了一种新型开环赛氏菌缩肽(serratamolide)类似物的结构。但该方法不能有效筛选目标途径相关分子簇,且方法注释能力主要依赖分子簇内可搜库注释的种子节点。植物次生代谢物结构存在大量修饰基团,在MS/MS中常以中性丢失形式体现^[17]^。植物代谢途径数据库包含大量代谢反应信息,如植物代谢途径(Plant Metabolic Pathways,PlantCyc,https://www.plantcyc.org/)^[18]^收录了5234个反应,京都基因与基因组百科全书(Kyoto Encyclopedia of Genes and Genomes, KEGG, https://www.genome.jp/kegg/)收录了11744个生化反应。将代谢途径中的修饰反应用于非靶向数据中代谢特征的筛选,可有助于提高途径代谢物的发现效率^[19]^。

为此,本研究针对次生代谢物种类多、结构复杂,且存在大量未知结构代谢物的分析挑战,以玉米叶片苯丙烷途径代谢物为例,利用代谢途径的修饰反应以及现有质谱数据库,发展用于非靶向LC-HRMS/MS代谢组数据中重要途径代谢物筛选和注释的新方法,为次生代谢物的注释和途径解析提供新思路。

## 1 实验部分

### 1.1 仪器、试剂与材料

ACQUITY UPLC超高效液相色谱系统购自美国Waters公司。Triple TOF 5600+飞行时间质谱(TOF-MS)购自美国AB SCIEX公司。超纯水由购自美国Billerica公司的Milli-Q系统纯化制备。乙腈(HPLC级)和甲醇(HPLC级)购自德国Merck公司。甲酸(纯度98%)和碳酸氢铵购自中国J & K Scientific有限公司。

### 1.2 样品预处理

玉米叶片代谢组提取:准确称取玉米新鲜叶片组织冻干粉50 mg,置于1.5 mL离心管中。加入1.0 mL 80%(v/v)甲醇水提取剂,涡旋提取5 min,在4 ℃条件下,以21500 g转速离心10 min。取700 μL上清液,放入真空离心浓缩仪冻干。冻干样品加入100 μL 80%(v/v)甲醇水复溶,复溶液在4 ℃条件下,以21500 g转速离心10 min。取上清液用于仪器分析。

### 1.3 非靶向代谢组学分析

正离子模式下的液相色谱条件 Waters ACQUITY BEH C_18_色谱柱(100 mm×2.1 mm, 1.7 μm);柱温:50 ℃;进样器温度:4 ℃;流动相A: 0.1%(v/v)甲酸水溶液;流动相B: 0.1%(v/v)甲酸乙腈溶液;流速:0.35 mL/min;进样量:5 μL。洗脱梯度:0~1.0 min, 5%B; 1.0~24.0 min, 5%B~100%B; 24.0~28.0 min, 100%B; 28.0~28.1 min, 100%B~5%B; 28.1~30.0 min, 5%B。

负离子模式下的液相色谱条件 Waters ACQUITY HSS T_3_色谱柱(100 mm×2.1 mm, 1.8 μm);柱温:50 ℃;进样器温度:4 ℃;流动相A: 6.5 mmol/L碳酸氢铵水溶液;流动相B: 含6.5 mmol/L碳酸氢铵的95%(v/v)甲醇水溶液;流速:0.35 mL/min;进样量:5 μL。洗脱梯度:0~1.0 min, 2%B; 1.0~18.0 min, 2%B~100%B; 18.0~22.0 min, 100%B; 22.0~22.1 min, 100%B~2%B; 22.1~25.0 min, 2%B。

质谱条件 离子源:电喷雾电离(ESI)源,采用正、负离子模式检测;扫描方式:一级全扫描质量范围*m/z* 50~1250;二级数据依赖型扫描(Top 15)质量范围*m/z* 50~1250;碰撞能量:15、30和45 eV;离子源温度:500 ℃;电喷雾电压:4500 V;气帘气压强:0.241 MPa;雾化气(gas 1)压强:0.345 MPa;加热气(gas 2)压强:0.345 MPa。

### 1.4 数据处理

#### 1.4.1 原始数据预处理

采用MarkerView 1.2.1软件处理质谱原始数据,获得包含代谢特征保留时间、质荷比和峰强度的质谱峰列表。采用ProteoWizard 3.0.10240软件将质谱原始数据文件转化为. mgf格式的二级质谱文件。

#### 1.4.2 修饰代谢组筛选

首先,从代谢途径数据库收集修饰反应,将修饰类型的名称、分子式和精确相对分子质量整理至.csv格式的文件中,生成修饰类型列表。将非靶向代谢组数据的质谱峰列表、二级质谱文件,以及修饰类型列表作为输入文件,运行自编程序包ModifiedMetMRM。设置质谱峰列表与二级质谱文件的匹配参数为质量精度15×10^-6^,保留时间窗口12 s。基于修饰类型列表获取非靶向代谢组数据中修饰代谢组的匹配参数为质量窗口15×10^-6^,子离子最低绝对强度为100。离子融合参数为质量窗口15×10^-6^,保留时间窗口12 s。

#### 1.4.3 探针分子数据库构建

下载GNPS(https://gnps.ucsd.edu/)中的3个质谱数据集(GNPS Library, NIH Natural Products Library Round 1和NIH Natural Products Library Round 2)。采用自编代码收集3个数据库化合物的SMILES,并在ChemDes平台(http://www.scbdd.com/convert/convert/)将其转化为InChIKey。利用ClassyFire(https://cfb.fiehnlab.ucdavis.edu/)工具获得化合物的化学分类,保留其中苯丙烷类化合物,获取化合物的相关信息,包括采集MS/MS的仪器类型、SMILES和二级质谱图等。

#### 1.4.4 分子网络构建

分子网络由开源工具GNPS平台完成。建网参数:不少于6个子离子匹配,MS/MS相似度阈值为0.7;母离子质量精度阈值:0.01 Da;子离子质量精度阈值:0.02 Da;单个连通网络节点数最大值:500;单个节点最大相邻节点数:50。分子网络可视化由软件Cytoscape 3.8.0实现。

#### 1.4.5 代谢物定性

采用基于SMRT数据集构建的GNN-RT模型^[20]^,通过标准品迁移学习预测本实验色谱条件下的保留时间;使用开源工具CFM-ID 4.4.3 (https://hub.docker.com/r/wishartlab/cfmid)预测代谢物的虚拟(*in silico*)二级质谱;二级质谱相似度计算采用谱熵算法^[21]^。代谢物化学类别预测采用SIRIUS 4^[22]^中的CANOPUS^[12]^完成,使用默认参数。

## 2 结果与讨论

### 2.1 方法框架

方法总体框架如图1所示,途径代谢物的筛选与注释步骤主要包括:1)基于公共代谢途径数据库及文献报道,收集参与途径的修饰反应,构建修饰基团质谱数据库,进而从非靶向实验数据中筛选修饰代谢组(含有修饰基团的代谢物); 2)从开源质谱数据库中收集目标途径代谢物及其类似物作为探针分子,构建探针分子串联质谱数据库;3)将探针分子与修饰代谢组共建分子网络;4)筛选目标途径代谢物分子簇;5)基于探针分子结构、修饰基团、子结构信息等注释代谢物。

**图 1:**
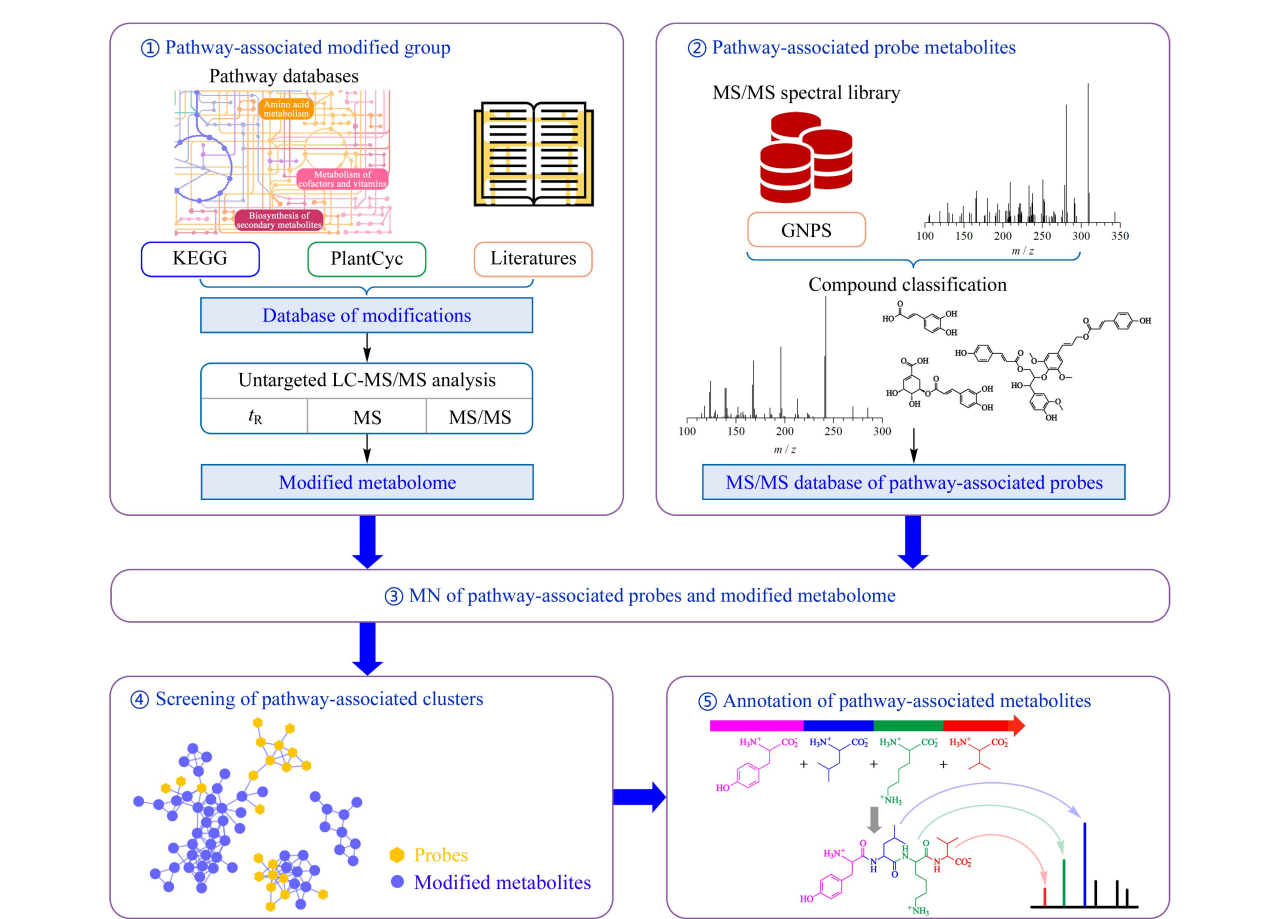
重要途径代谢物的筛选与注释流程

### 2.2 修饰代谢组筛选

利用开源代谢途径知识库,如:KEGG、PlantCyc以及文献报道^[23⇓-25]^,收集参与苯丙烷途径的代谢反应,提取修饰基团。表1给出了收集到的61种修饰反应类型,包括11种通用修饰类型(甲基化、甲氧基化、羟基化、甲氨基化、乙酰化、羧基化、硫酸化、丙二酰化、戊糖基化、脱氧己糖基化和己糖基化)和50种途径特异性修饰类型(13种胺结合、5种羟基肉桂酰结合、13种酸结合、18种醇结合和1种异戊二烯化修饰)。

**表 1:** 参与苯丙烷代谢的后修饰反应

Modified type	Neutral loss	Generic or specific	Modified type	Neutral loss	Generic or specific
Methylation	CH_2_	generic	Phenylacetyl coupling	C_8_H_6_O	specific
Methoxylation	CH_2_O	generic	Hydroxybenzoate	C_7_H_4_O_2_	specific
Hydroxylation	H_2_O	generic	p-Hydroxybenzoylation	C_7_H_6_O_2_	specific
Methyl ammonia	CH_3_NH_2_	generic	Tartarate	C_4_H_4_O_5_	specific
Acetylation	CH_2_CO	generic	Dihydroxybenzoic acid coupling	C_7_H_4_O_3_	specific
Carboxylation	COO	generic	Hydroxyadipic acid coupling	C_6_H_8_O_4_	specific
Sulfation	SO_3_	generic	Vanillate	C_8_H_6_O_3_	specific
Malonyl	C_3_H_2_O_3_	generic	3-Dehydroshikimic acid coupling	C_7_H_6_O_4_	specific
Pentosylation	C_5_H_8_O_4_	generic	Shikimic acid coupling	C_7_H_8_O_4_	specific
Deoxyhexosylation	C_6_H_10_O_4_	generic	Quinic acid coupling	C_7_H_10_O_5_	specific
Hexosylation	C_6_H_10_O_5_	generic	Syringate	C_9_H_8_O_4_	specific
Putrescine	C_4_H_12_N_2_	specific	Glycerol	C_3_H_6_O_2_	specific
Cadaverine	C_5_H_14_N_2_	specific	Quinol	C_6_H_4_O	specific
Agmatine	C_5_H_14_N_4_	specific	Hydroxybenzyl alcohol	C_7_H_6_O	specific
Tyramine	C_8_H_11_NO	specific	Hydroxyquinol	C_6_H_4_O_2_	specific
Spermidine	C_7_H_19_N_3_	specific	Vanillyl alcohol	C_8_H_8_O_2_	specific
Octopamine/dopamine	C_8_H_11_NO_2_	specific	Coumaryl alcohol	C_9_H_8_O	specific
Tryptamine	C_10_H_12_N_2_	specific	Caffeyl alcohol	C_9_H_8_O_2_	specific
3-Methoxytyramine	C_9_H_13_NO_2_	specific	Coniferyl alcohol	C_10_H_10_O_2_	specific
Noradrenaline	C_8_H_11_NO_3_	specific	5-OH-Feruloyl alcohol	C_10_H_10_O_3_	specific
Serotonin	C_10_H_12_N_2_O	specific	Sinapyl alcohol	C_11_H_12_O_3_	specific
3'-Methoxyoctopamine	C_9_H_13_NO_3_	specific	Non-condensed vanillyl alcohol	C_8_H_10_O_3_	specific
5-Methoxytryptamine	C_11_H_14_N_2_O	specific	Non-condensed coumaryl alcohol	C_9_H_10_O_2_	specific
Spermine	C_10_H_26_N_4_	specific	Non-condensed caffeyl alcohol	C_9_H_10_O_3_	specific
Coumaryl	C_9_H_6_O_2_	specific	Non-condensed coniferyl alcohol	C_10_H_12_O_3_	specific
Caffeoyl	C_9_H_6_O_3_	specific	Non-condensed 5-OH-feruloyl alcohol	C_10_H_12_O_4_	specific
Feruloyl	C_10_H_8_O_3_	specific	Non-condensed sinapyl alcohol	C_11_H_14_O_4_	specific
5-OH-Feruloyl	C_10_H_8_O_4_	specific	Dimethoxyquinol	C_8_H_8_O_3_	specific
Sinapyl	C_11_H_10_O_4_	specific	Syringyl alcohol	C_9_H_10_O_3_	specific
Malate	C_4_H_4_O_4_	specific	Isoprenylation	C_5_H_8_	specific
Glyceric acid coupling	C_3_H_4_O_3_	specific			

从玉米叶片正、负离子模式非靶向代谢组学数据中筛选到60种修饰类型,对应1385个和1412个代谢物。其中,正、负离子模式下分别有32.27%(447/1385)和26.06%(368/1412)的代谢物含有两种及以上修饰基团,通用修饰类型占比32.31%(正离子模式)和34.85%(负离子模式);正离子模式下,特异性修饰类型主要是胺结合(7.76%)、羟基肉桂酰结合(8.09%)、酸结合(13.91%)和醇结合(36.28%)修饰;异戊二烯化修饰占比较低,仅为1.66%。负离子模式与正离子模式类似,上述特异性修饰及异戊二烯化修饰的占比分别为3.94%、13.62%、19.21%、28.12%和0.26%。

### 2.3 探针分子串联质谱数据库构建

基于ClassyFire^[26]^化学分类从GNPS数据库中包含天然产物较多的3个质谱数据集(GNPSLibrary、NIH Natural Products Library Round 1和NIH Natural Products Library Round 2)中共收集到1542个苯丙烷类化合物的正离子模式二级谱图2677张和661个苯丙烷类化合物的负离子模式二级谱图814张,建立探针分子串联质谱数据库。图2给出了探针分子二级质谱采集条件统计,其中来自飞行时间质谱的二级谱图占主要的比重,正、负离子模式占比分别为83.75%和97.67%(见图2a)。对探针分子的化学类别进行统计,分属36个亚类,其中类黄酮、异黄酮、香豆素及其衍生物、肉桂酸及其衍生物占比较大,正、负离子模式占比分别为68.61%和71.26%(见图2b)。

**图 2:**
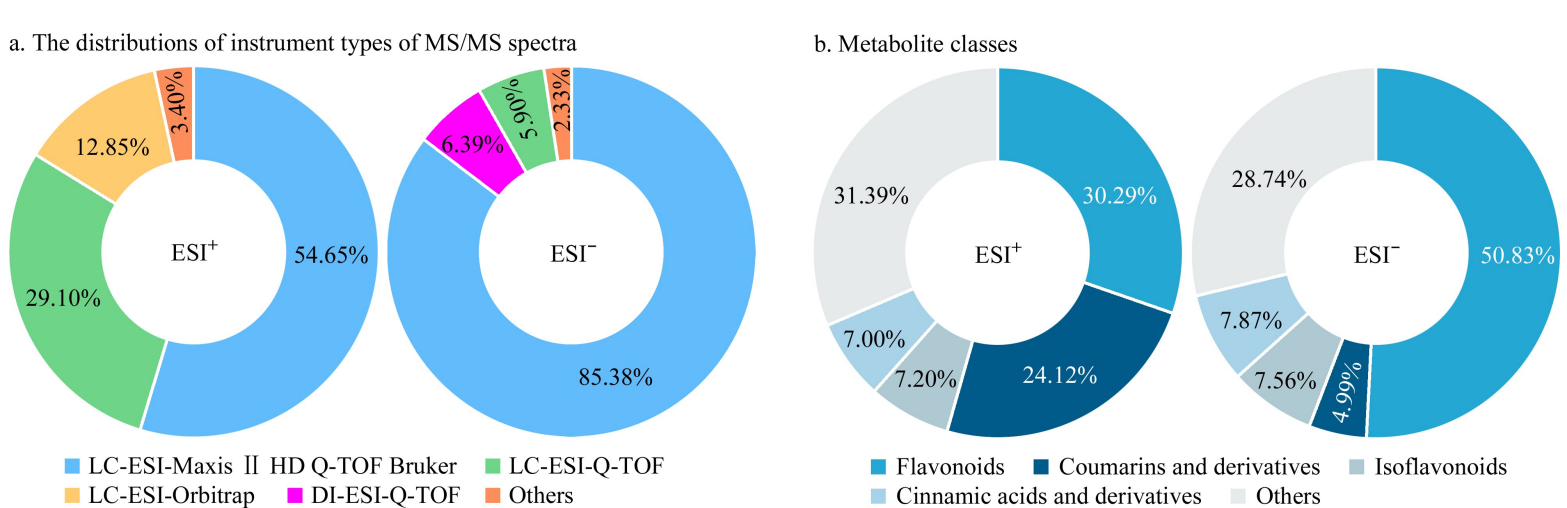
探针分子串联质谱数据库收录统计

### 2.4 途径代谢物筛选

将探针分子与玉米叶片中筛选出的修饰代谢组共建分子网络,选取其中苯丙烷途径代谢物所在的分子簇。分子簇筛选条件为:簇内同时含有探针分子与修饰代谢物,或虽不含探针分子但含有一个及以上途径特异性修饰的代谢物。满足筛选条件的分子簇中共含有392(正离子模式)和417个(负离子模式)修饰代谢物。图3a所示为负离子模式筛选出的分子簇,簇内的探针分子可快速提示簇内代谢物所属的途径信息。如图3b所示,簇内8个探针分子(黄色)均为木脂素类化合物,提示该分子簇为木脂素及其结构类似物。仅含途径特异性修饰基团的代谢物分子簇如图3c,簇内含有木脂素途径特异性醇结合的修饰基团,如松柏醇(non-condensed coniferyl alcohol)和芥子醇(non-condensed sinapyl alcohol)等,该分子簇也被快速识别为木脂素途径代谢物。

**图 3:**
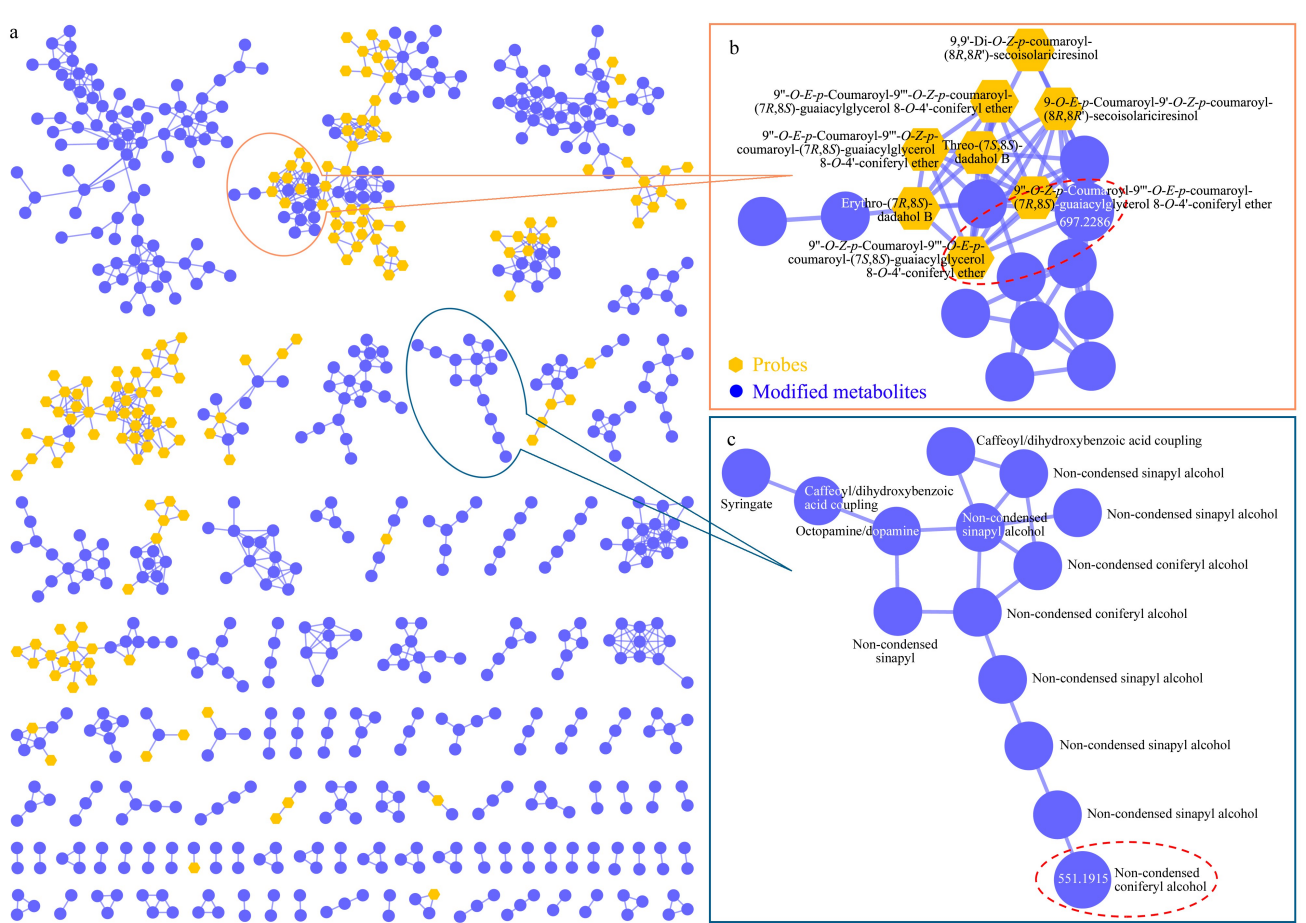
基于探针-修饰组分子网络筛选出的苯丙烷途径代谢物

### 2.5 途径代谢物注释

对筛选出的途径代谢物进行结构注释,对于含有探针分子的分子簇,以探针分子为初始种子节点,结合修饰基团信息进行网络传播注释。以图3b红色虚框中紫色节点(*m/z* 697.2286)的注释为例,其相邻黄色节点为探针分子9″-*O-Z-p*-香豆酰-9″'-*O-E-p*-香豆酰-(7*S*,8*S*)-愈创木酰甘油8-*O*-4'-松柏醚(9″-*O-Z-p*-coumaroyl-9″'-*O-E-p*-coumaroyl-(7*S*,8*S*)-guaiacylglycerol 8-*O*-4'-coniferyl ether, *m/z* 667.2180);它们之间存在Δ*m/z*为30.0105的质量差,推断其为探针分子甲氧基化修饰的产物。此外,该节点只有香豆酰(coumaroyl)一种修饰(中性丢失),说明甲氧基化修饰未发生在香豆酰部分。进一步从该节点的二级谱图可知,香豆酰(146.0357)以中性形式丢失后,产生子离子*m/z* 551.1929,它与子离子*m/z* 341.1012之间可能存在*m/z* 210.0917的中性丢失,从表1可知归属为非缩合芥子醇(non-condensed sinapyl alcohol),基于该子结构推断甲氧基化修饰仅能发生在阿魏醇(feruloyl alcohol)上。对其二级质谱碎片离子进行子结构注释(见图4a),并将碎片信息进行化合物结构拼接,将得到的结构搜索PubChem数据库,确定为稀有木脂素A(dadahol A)。

**图 4:**
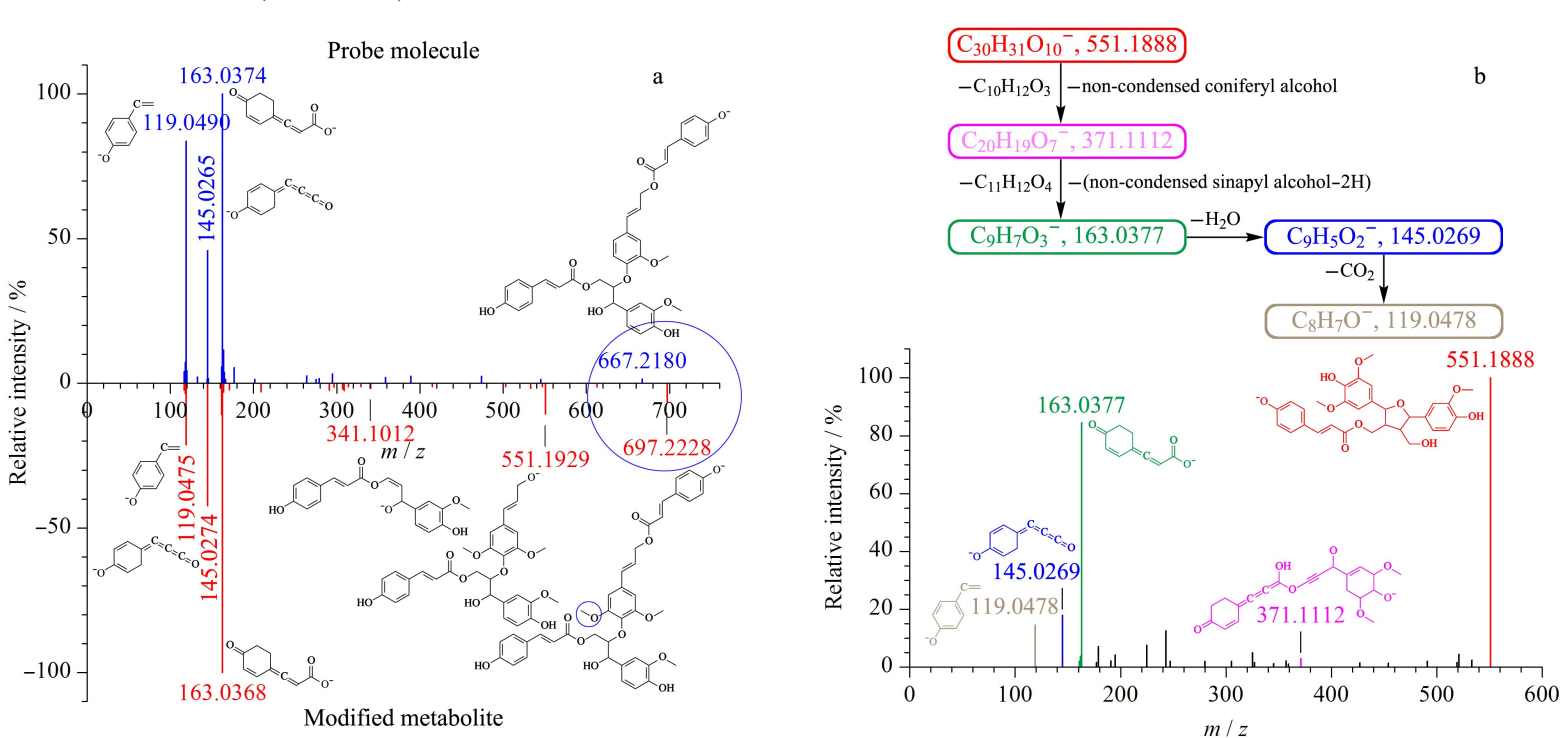
(a)基于已知相邻节点的代谢物传播注释和(b)代谢物从头注释

对只含有途径特异性修饰代谢物的分子簇,如簇内有可被搜库鉴定的节点,则以注释节点为初始种子,注释过程与含有探针分子的分子簇类似。对于无相邻注释节点的修饰代谢物采用从头注释方式,如图3c所示簇内代谢物仅有修饰基团提供的子结构信息。以簇末端代谢物(*m/z* 551.1915)的结构解析为例说明如下:该代谢物含有非缩合松柏醇(non-condensed coniferyl alcohol)子结构,经中性丢失(*m/z* 180.0786)产生*m/z* 371.1112的子离子;另外还有香豆酸(coumaric acid)特征离子(*m/z* 163.0377),它与子离子*m/z* 371.1112之间可能存在*m/z* 208.0735的中性丢失;该中性丢失与修饰基团库中的非缩合芥子醇(*m/z* 210.0892)相差2.0157,推断可能是非缩合芥子醇开环失去2个H;将得到的子结构非缩合松柏醇、香豆酸和非缩合芥子醇进行拼接,符合二级谱图注释结果的仅存在一种合理结构(见图4b)。该结构经PubChem和SciFinder数据库搜索,均未见收录。采用上述注释方法,将正、负离子均注释出的同一代谢物,根据其结构去冗余后共初步注释出129个苯丙烷途径代谢物,其中89个在PubChem和SciFinder数据库中已有收录,26个为数据库未报道的“未知结构”化合物。采用注释代谢物的MS/MS对其结构进一步验证,其中68个注释代谢物的化合物类别可被准确预测为苯丙烷代谢物。对其中115个有确切结构的代谢物(其余14个为同分异构体)预测其保留时间,其中102个代谢物的预测保留时间相对误差小于30%。采用CFM-ID工具预测了115个代谢物的二级谱图,并计算了与实验二级谱图的相似性;其中,具有较高相似性(>0.5)的代谢物有28个,较相似(0.4~0.5)的代谢物有30个,有一定相似性(<0.4)的有57个。此外,对其中10个有标准品的代谢物进行了验证,验证结果显示注释结构正确。

从注释结果可知,129个苯丙烷途径代谢物涉及苯丙烷下游主要分支途径产生的次生代谢物(见图5),如黄酮分支途径的8个类黄酮、19个氧苷类黄酮、32个碳苷类黄酮,羟基肉桂酸途径的31个羟基肉桂酸酰胺及其衍生物,以及木脂素合成途径的22个(新)木脂素/木脂素苷等。其中4个氧苷类黄酮、4个碳苷类黄酮、6个羟基肉桂酸酰胺及其衍生物和11个木脂素结构在PubChem和SciFinder数据库中均未被收录(见图5括号内红色数字)。

**图 5:**
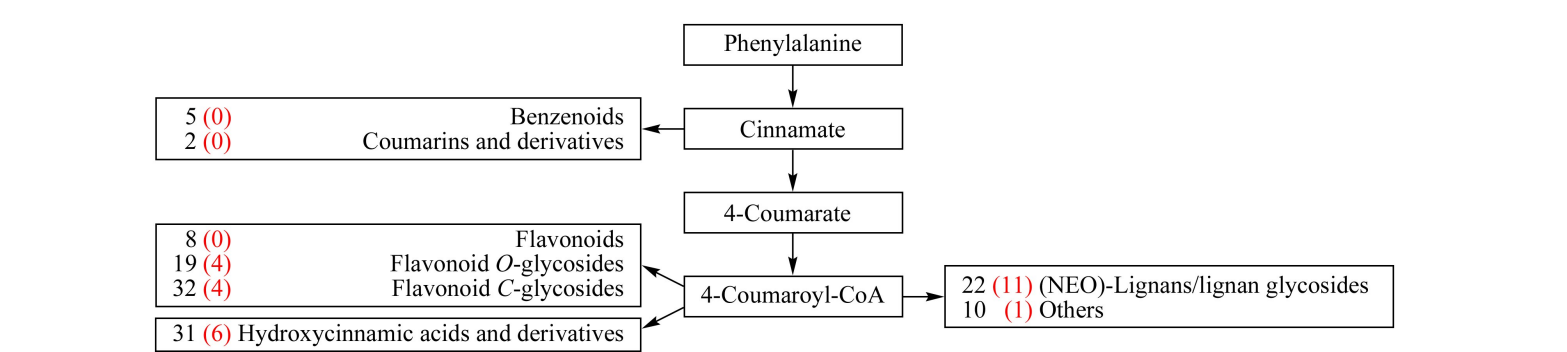
注释代谢物的分支途径分布

## 3 结论

本研究以苯丙烷途径代谢物的筛选和注释为例,发展了一种基于修饰组和探针分子的重要途径代谢物高效筛选和注释方法。仅从一种玉米叶片组织中就注释出了129个苯丙烷途径代谢物,其中有26个未被PubChem和SciFinder数据库收录,显示了方法在发现与注释途径代谢物特别是未在数据库收录的“未知结构”代谢物方面的能力。鉴于目前途径数据库提供的修饰反应尚不全面,以及开源质谱数据库因谱图质量、仪器采集条件差异等原因,方法注释能力还未能充分发挥。随着数据库信息的不断积累和完善,以及基于机器学习的子结构预测方法不断成熟,未来将在重要代谢途径挖掘和利用方面发挥更大的作用。
